# Prognostic and predictive value of tumor-infiltrating lymphocytes in breast cancer: a systematic review and meta-analysis

**DOI:** 10.1007/s12094-015-1391-y

**Published:** 2015-10-12

**Authors:** X. Yu, Z. Zhang, Z. Wang, P. Wu, F. Qiu, J. Huang

**Affiliations:** Cancer Institute (Key Laboratory of Cancer Prevention and Intervention, National Ministry of Education, Provincial Key Laboratory of Molecular Biology in Medical Sciences), The Second Affiliated Hospital, Zhejiang University School of Medicine, Hangzhou, 310009 China; Department of Oncology, Second Affiliated Hospital, Zhejiang University School of Medicine, Hangzhou, 310009 China; Department of Gynecology, Second Affiliated Hospital, Zhejiang University School of Medicine, Hangzhou, 310009 China

**Keywords:** Tumor-infiltrating lymphocytes, Breast cancer, Clinicopathological features, Prognosis

## Abstract

**Background:**

Breast cancer is the most common invasive cancer to affect women in the world. Studies showed tumor-infiltrating lymphocytes can exhibit both beneficial and harmful effects on the biology and clinical outcome of breast cancer, the conclusion still remains incomplete. Here, we conducted a meta-analysis to evaluate the relationship between tumor-infiltrating lymphocytes and breast cancer.

**Methods:**

A comprehensive search strategy was used to search relevant literatures in PubMed and the ISI Web of Science. The correlation among TILs and breast cancer clinicopathological features and prognosis was analyzed by using Review Manager 5.3 and Stata 12.0.

**Result:**

Seventeen eligible studies consisting of 12,968 participants were included. We found that higher value of tumor-infiltrating lymphocytes had no relationship with breast cancer clinicopathological variables. Interestingly, it was correlated with response to neoadjuvant chemotherapy in majority (pooled RR 2.43, 95 % CI 1.99–2.97). Moreover, higher value of total tumor-infiltrating lymphocytes (both intraepithelial and stromal) was associated with better prognosis (pooled HR 0.88, 95 % CI 0.83–0.94), whereas some subtypes predicted a worse prognosis.

**Conclusion:**

This meta-analysis indicated that high value of total TILs is not associated with breast cancer clinicopathological features, but can predict a favorable outcome for neoadjuvant chemotherapy in majority except for hormone receptor (−) subtype. And higher total TILs (both intraepithelial TILs and stromal TILs) may be the potential better prognostic indicators, while some subtypes like PD-1^+^ TILs and Foxp3^+^ TILs show a worse prognosis.

**Electronic supplementary material:**

The online version of this article (doi:10.1007/s12094-015-1391-y) contains supplementary material, which is available to authorized users.

## Introduction

Breast cancer is one of the most common invasive cancers to affect women health in the world. In 2013, it has accounted for 29 % of all new cancer cases and 14 % of all cancer deaths, becoming the second highest cause of cancer death in women after lung cancer [1, 2]. Nonetheless, due to the understanding of the breast cancer biology and improvement in early diagnosis and treatment, its mortality has steadily declined. Currently, accumulating evidences have shown that the malignancy and biological features of cancer depend on its genetic abnormalities as well as the interplay between cancer cells and microenvironment. Conversely, conventional researches concerned more about the biological features and the prognosis based on the indicators of breast cancer itself, such as histological grade, expression of the hormone receptors (estrogen receptor and progesterone receptor), proliferation marker-Ki67, and amplification status of the HER2 gene et al. [3, 4]. However, recent studies have reported that the tumor microenvironment, comprising adipocytes, tumor-associated fibroblasts, immune cells, extracellular matrix, cytokines and other factors, also plays an important role in tumor formation, growth, invasion and metastasis. Immune cells, like tumor-associated macrophages (TAMs), studies indicated that TAMs generally play a protumoral role, and in the primary tumor, TAMs can stimulate angiogenesis and enhance tumor cell invasion, motility, and intravasation [5, 6]. Clinical evidences indicate the association between high TAMs influx and poor prognosis in breast cancer patients [7, 8]. Also, tumor-infiltrating lymphocytes (TILs) have distinct roles in modulation of the tumor niche, favoring or inhibiting carcinogenesis and cancer progression. Moreover, some subtypes of lymphocytes secrete IL-6 and IL-8, which in turn activate PI3K/AKT, NF-κB, STAT3 signaling, and generate a positive feedback loop between the tumor cells and immune microenvironment [9, 10].

Limited data showed that total TILs were associated with a better prognosis [11, 12]. In breast cancer, TILs existence before chemotherapy is a good phenomenon, which prompts the therapeutic effect of neoadjuvant therapy [13, 14]. But the type, density, and location of TILs in breast cancer exhibit different values for assessing disease prognosis and progression. The majority of TILs are prominent CD8^+^ T cells, which are the major effector cell type, and have been linked to a better prognosis [15]. However, Foxp3^+^ T cells or PD-1^+^ T cells infiltration mediates tumor immune escape and reminds a worse prognosis [16]. Thus, subtypes of TILs can exert both inhibitory and stimulatory effects on breast cancer and the prognostic value of TILs remains complex and controversial. To address this controversy, we conducted a meta-analysis aimed to evaluate the total or subtype of TILs as a potential prognostic marker for breast cancer and to determine the relationship between TILs and several clinicopathological features.

## Materials and methods

### Literature search

This systematic review and meta-analysis were conducted according to the Preferred Reporting Items for Systematic Reviews and Meta-Analyses (PRISMA) statement [17]. A systematic literature search for the following tags: “tumor-infiltrating lymphocytes and breast cancer” or “tumor-associated lymphocytes and breast cancer”-related papers in the electronic databases PubMed and the Web of Science from January 1990 to July 2014 was conducted independently by two investigators. The citation lists associated with the studies, including review articles that were retrieved in the search, were used to identify additional relevant publications. The title and abstract of each study identified in the search were scanned to exclude any clearly irrelevant reports.

### Selection criteria

The studies included in this meta-analysis were either randomized controlled studies (RCTs) or observational studies (case–control or cohort) that evaluated the association between TILs and breast cancer. The criteria for inclusion were as follows: (a) articles evaluating the relationship between TILs and parameters such as clinicopathological features and prognostic factors of breast cancer; (b) articles containing sufficient published data to determine an estimate of relative risk (RR) or hazard ratio (HR) and a 95 % confidence interval (95 %CI); and (c) full-text, original research articles published in English.

The following studies were excluded: (1) overlapping articles or duplicate data; (2) non-English languages; (3) the types of reviews, comments or letters; (4) articles published in books; and (5) lacking information.

### Data extraction

Data from eligible studies were independently extracted in a standardized manner by the two investigators. Disagreements in data extraction were resolved by consensus and by referring back to the original article. The following data were obtained from each article: first author’s last name; year of publication; country of the population studied; number of participants; duration of follow-up; the choice of cutoff scores for the definition of positive staining or staining intensity; T category (T0–1, T2–4); N category; HER-2, hormone receptor status; and most importantly, pathological complete response (pCR) rate, the 5-year overall survival (OS) and disease-free survival (DFS) rates.

The cutoff value for the TILs varied among studies, we defined higher expression of TILs value according to the original articles. And high TILs were defined as higher value of total TILs on hematoxylin and eosin (H&E)-stained sections. The T category was determined according to the American Joint Committee on Cancer (AJCC) cancer staging manual (one group T0–1, other group T2–4). We defined hormone receptor positive either ER ≥1 % or PR ≥1 % under immunohistochemistry (IHC), and classified HER-2 positive if HER-2 gene amplification using in suit hybridization(ISH) or scored as 3+ by IHC method. To avoid bias from studies contributing very long-term follow-up data compared with other studies, both OS and DFS rates were standardized to include 5 years of follow-up in all studies. For the articles that did not provide 5-year OS and DFS rates directly, Kaplan–Meier curves were evaluated using GetData Graph Digitizer 2.24 (http://getdatagraph-digitizer.com).

### Assessment of study quality

The qualities of 17 eligible studies included in our meta-analysis were assessed according to the Newcastle–Ottawa scale (NOS). The NOS contains eight items, which are categorized into the three dimensions of selection, comparability, and outcome (cohort studies) or exposure (case–control studies). The quality scores in NOS ranged from 0 (lowest) to 9 (highest) and studies with scores 6 or more were rated as high quality. All included studies obtained scores of 6 or more in the methodological assessment, indicating that they were of high quality (Table 1).

**Table 1:** Characteristics of the included studies

Source	Year	Type of lymphocytes	Country	Number of participant	Duration of follow-up (months)	Clinicopathologic characteristic	Short-term prognosis	Long-term prognosis	Quality score
Makiko Ono [33]	2012	Total types	Japan	180	64.8	HR, HER-2	pCR	DFS	7
Sherene Loi [44]	2013	Total types	Hungary	2009	96	–	–	OS, DFS	5
S. Loi [27]	2014	Total types	Australia	1010	–	G, HR, HER-2	–	–	4
M. V. Dieci [32]	2014	Total types	France	278	76	G, T, N	–	OS, MFS	8
Rin Yamaguchi [41]	2011	Total types	Japan	68	–	–	pCR	–	6
Carsten Denkert [17]	2010	Total types	Germany	1058	–	–	pCR	–	7
Sylvia Adams [40]	2014	Total types	USA	705	127	–	–	OS, DFS	7
Hee Jin Lee [42]	2013	Total types	Korea	175	–	–	pCR	–	6
Nathan R. West [43]	2011	Total types	Canada	113	–	–	pCR	–	6
S. Muenst [34]	2013	PD-1^+^	USA	660	65	G, T, N, HR, HER-2, Ki67	–	OS	7
Shenyou Sun [35]	2013	PD-1^+^, Foxp3^+^	China	208	72	G, T, N, HR, HER-2	–	OS, DFS	7
Chunling Ma [19]	2012	CD8^+^, Foxp3^+^, γδT	China	81	60	T, N, HR, HER-2	–	OS, DFS	6
A. N. Seo [15]	2013	CD8^+^, Foxp3^+^	Korea	153	–	G, HR, HER-2, Ki67	–	–	5
Sahar M. A. Mahmoud [36]	2010	Foxp3^+^	UK	1445	180	T, N, HR, HER-2	–	CSS	6
Rafal Matkowski [45]	2009	CD8^+^	Poland	88	39	–	–	OS, DFS	5
Shuzhen Liu [38]	2012	CD8^+^	Canada	3403	65	G, T, N, HR, HER-2	–	CSS	6
Sahar M. A. Mahmoud [37]	2011	CD8^+^	Egypt	1334	127	G, HR, HER-2	–	CSS	6

*OS* overall survival, *DFS* disease-free survival, *CSS* cancer-specific survival, *MFS* metastasis-free survival, *pCR* pathologic complete response, *HER-2* human epidermal growth factor receptor-2, *Ki67* proliferating antigen Ki67, *PD-1* programmed death 1, *G* tumor grade, *T* tumor category, *N* lymph node category, *HR* hormone receptor, including progesterone and estrogen receptor

### Statistical analysis

This meta-analysis calculated the pooled RR or HR with its corresponding 95 % CI to assess the association of TILs with breast cancer using RevMan 5.3. Study heterogeneity was measured using the *Q* test and *I*^2^ test. Fixed-effects models (Mantel–Haenszel, *P* > 0.1 and *I*^2^ < 50 %) assume that the differences between the results of various studies are due to chance. Random-effects models (DerSimonian and Laird, *P* ≤ 0.1 or *I*^2^ ≥ 50 %) assume that the results can genuinely differ between studies. In the absence of heterogeneity, both fixed- and random-effects models provide similar results. When heterogeneity is present, the random-effects model is considered to be more appropriate than a fixed-effects model, resulting in wider intervals and a more conservative estimate of effect. The publication bias on the reported outcomes was assessed with the construction of contour-enhanced funnel plots and Egger’s tests; this analysis was performed by STATA version 12.0.

## Results

### Search results and characteristics of eligible studies

**Fig. 1:**
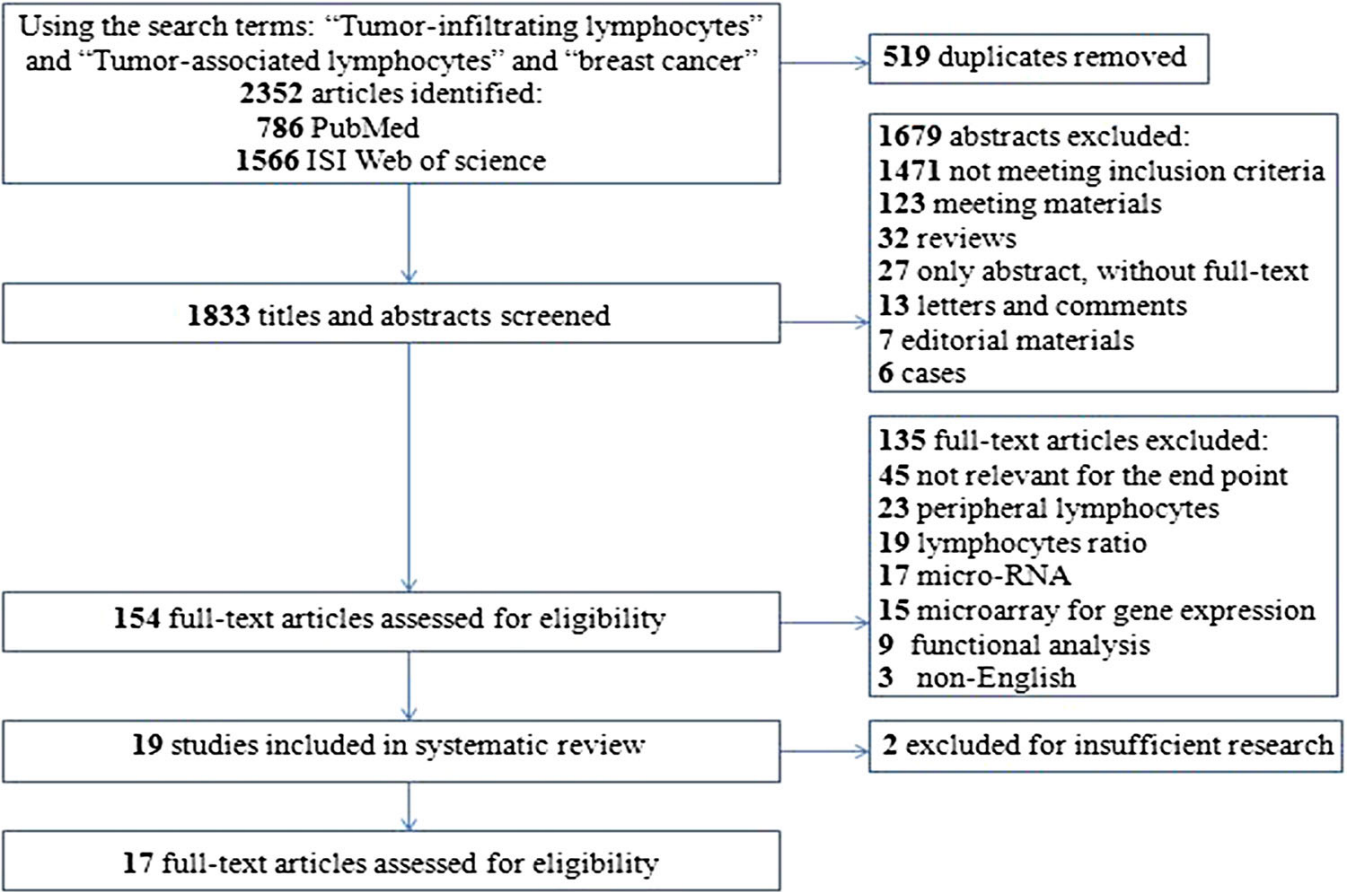
Flowchart of the selection of studies for inclusion in the meta-analysis

The detailed search steps are described in Fig. 1. Initially, 1833 potential articles were retrieved utilizing the search strategy described above. After titles and abstracts were reviewed, 1679 articles were excluded. Thus, 154 full-text papers was viewed, of these papers, another 135 were excluded because they did not provide data between TILs and clinicopathological features or specify whether disease-free survival (DFS)/overall survival (OS) rate was investigated. Finally, a total of 17 studies were included for the meta-analysis.

All features of the eligible studies in systematic review and meta-analysis are summarized (Table 1). These observational retrospective studies evaluated the level of TILs and clinicopathological features or prognostic parameters for breast cancer, consisting of approximately 12,968 participants with a median of 711 (from 68 to 3403) per study and with a median follow-up of 73 months (range 39–180). Among them, five were from the Europe, four from America, seven from Asia, and one from Africa, which covered the most areas around the world.

### Higher value of total TILs was not associated with breast cancer clinicopathological features, but some subtypes may have

High TILs value was not associated with certain clinical parameters of breast cancer, such as grade category (G1–2 vs. G3): (pooled RR 0.93, 95 % CI 0.77–1.13); hormone receptor status (+ vs. −): (pooled RR 0.48, 95 % CI 0.07–3.32); or HER-2 status (+ vs. −): (pooled RR 0.83, 95 % CI 0.61–1.12). So, we found that total TILs were not associated with tumor grade, hormone receptor or HER-2 status. Unfortunately, we had not analyzed the relationship of tumor size, lymph node status and Ki-67, due to the limited quantity of literatures (Supplementary Figure 1).

It is already known that TILs in breast cancer have several subtypes, such as CD8^+^ T cell, PD-1^+^ T cell and Foxp3+ T cell. Through the analysis, we found that PD-1^+^ TILs were related to high tumor grade (G1–2 vs. G3) (pooled RR 0.63, 95 % CI 0.54–0.73), big tumor size (T1 vs. T2–4) (pooled RR 0.72, 95 % CI 0.62–0.82), positive lymph node (+ vs. −) (pooled RR 1.76, 95 % CI 1.50–2.07), negative hormone receptor (+ vs. −) (pooled RR 0.75, 95 % CI 0.66–0.84) and HER-2 status (+ vs. −) (pooled RR 1.53, 95 % CI 1.08–2.16). Both Foxp3^+^ TILs and CD8^+^ TILs also had some relationships with breast cancer clinicopathological parameters (Table 2).

**Table 2:** Association between TILs and breast cancer clinicopathological feature

Subtype of TILs	Tumor grade (G1–2 vs. G3)			Tumor size (T1 vs. T2–4)			Lymph node status (+ vs. −)			Hormone receptor (+ vs. −)			HER-2 (+ vs. −)		
	RR (95 % CI)	*I* ^2^ (%)	*P* value	RR (95 % CI)	*I* ^2^ (%)	*P* value	RR (95 % CI)	*I* ^2^ (%)	*P* value	RR (95 % CI)	*I* ^2^ (%)	*P* value	RR (95 % CI)	*I* ^2^ (%)	*P* value
Total TILs	0.93 (0.77, 1.13)	0	0.46	–	–	–	–	–	–	0.48 (0.07, 3.23)	96	0.45	0.83 (0.61, 1.12)	0	0.22
PD-1^+^ TILs	0.63 (0.54, 0.73)	0	<0.00001	0.72 (0.62, 0.82)	0	<0.00001	1.76 (1.50, 2.07)	0	<0.00001	0.75 (0.66, 0.84)	0	<0.00001	1.53 (1.08, 2.16)	9	0.02
Foxp3^+^ TILs	0.60 (0.53, 0.68)	46	<0.00001	0.86 (0.71, 1.03)	47	0.11	1.31 (0.94, 1.81)	65	0.11	0.81 (0.72, 0.91)	56	0.0004	1.95 (1.09, 3.46)	71	0.02
CD8^+^ TILs	0.97 (0.73, 1.28)	92	0.81	0.97 (0.76, 1.24)	38	0.83	0.75 (0.33, 169)	91	0.48	0.92 (0.84, 1.00)	55	0.06	1.30 (1.12, 1.51)	0	0.0006

### Higher value of total TILs predicted a better response to neoadjuvant chemotherapy in most breast cancers, except hormone receptor negative ones

**Fig. 2:**
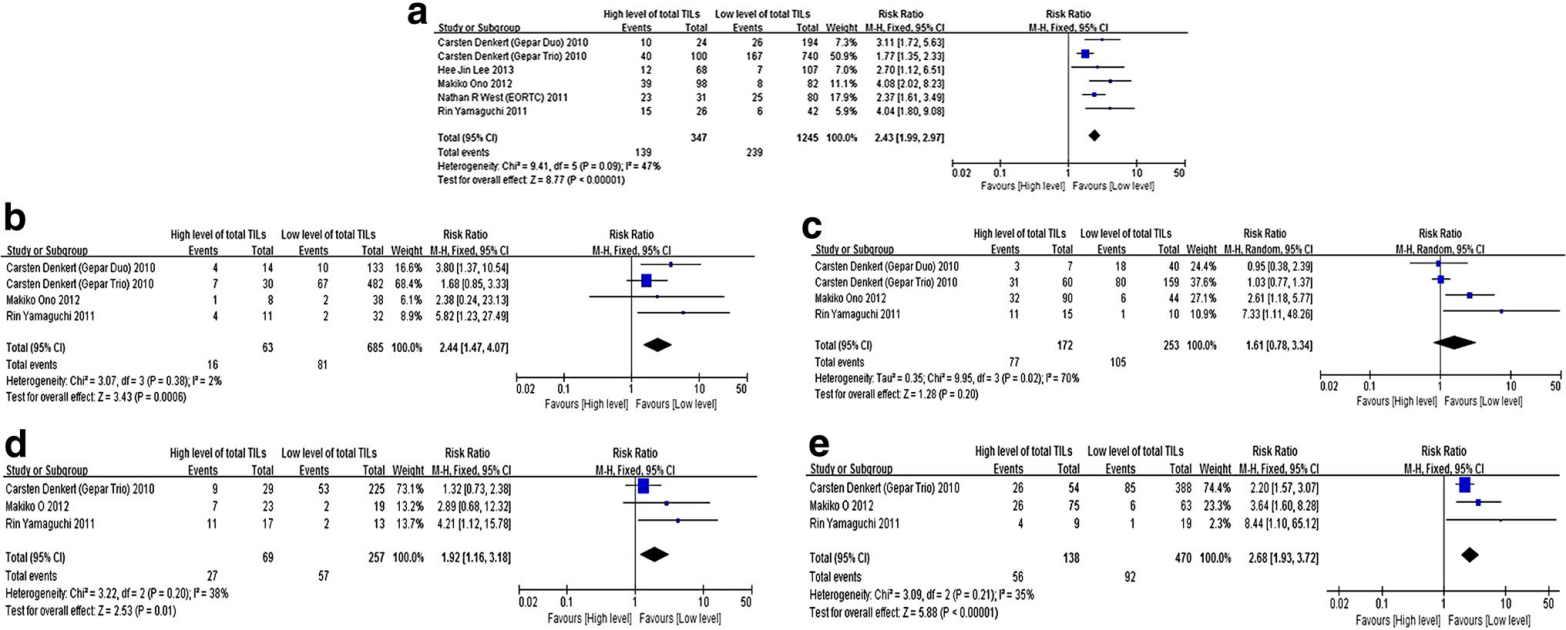
The forest plot of RRs was assessed for association between TILs and breast cancer short-term outcome (neoadjuvant chemotherapy pCR rate). **a** TILs and breast cancer, **b** TILs and hormone receptor (+) breast cancer, **c** TILs and hormone receptor (−) breast cancer, **d** TILs and HER-2 (+) breast cancer, **e** TILs and HER-2 (−) breast cancer

We defined the pathological complete response (pCR), which meant no residual invasive cancer cells in surgical specimens of primary tumor and axillary lymph node. Six studies containing 1970 patients were selected. The results showed over-expression of total TILs predicted a higher pCR rate (pooled RR 2.43, 95 % CI 1.99–2.97). Besides, high-TILs were also associated with elevated pCR rate for hormone receptor (+), HER-2 (+), and HER-2 (−) breast cancer, respectively (pooled RR were 2.24, 1.92, and 2.68, respectively) (Fig. 2).

### Higher value of total TILs was significantly associated with better prognosis, but several subtypes revealed a worse result

**Fig. 3:**
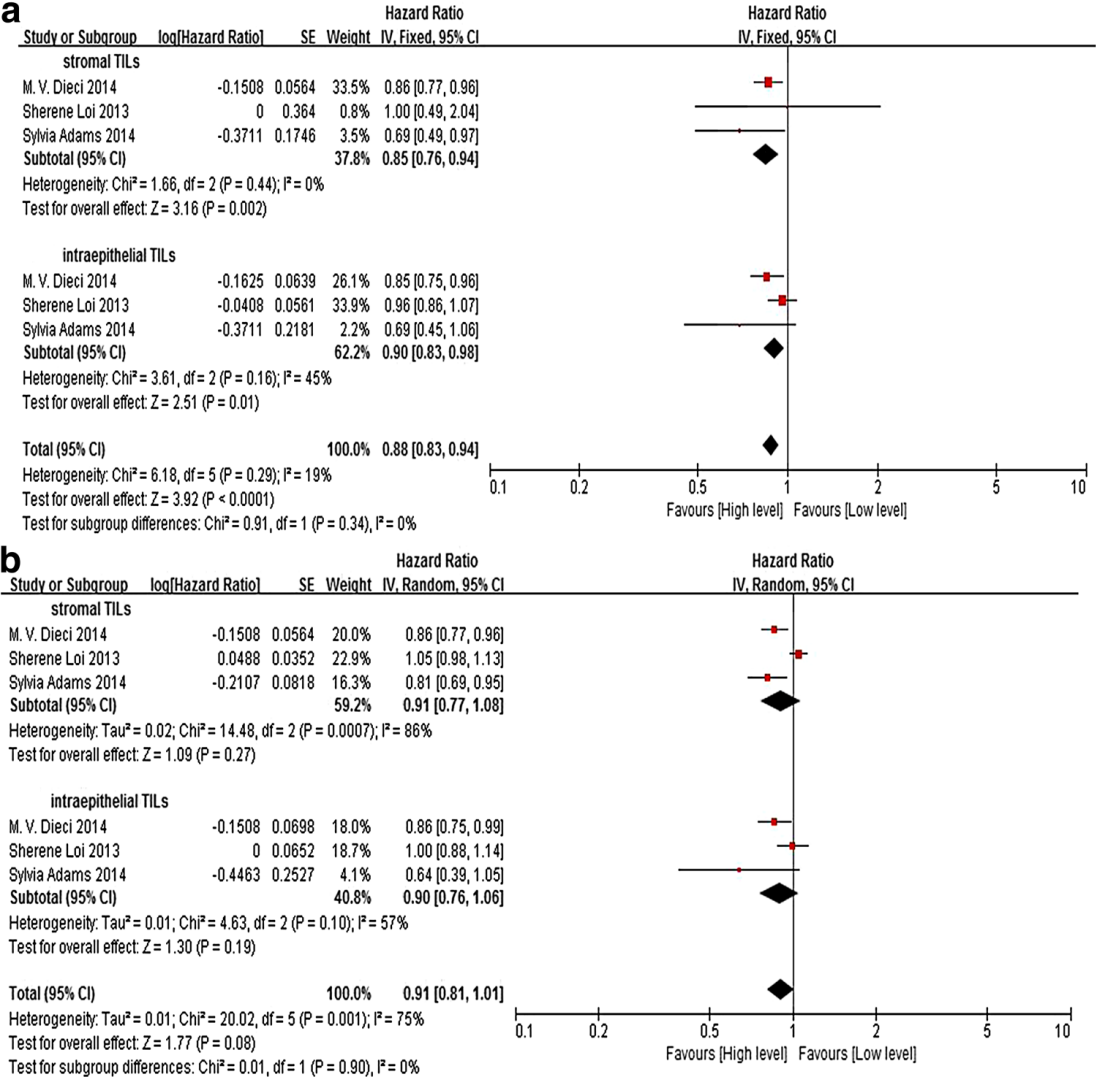
The forest plot of HRs was assessed for association between total TILs and breast cancer long-term prognosis. **a** Total TILs and disease-free survival/metastasis-free survival, **b** total TILs and overall survival

Three studies (including a total of 2992 patients) that demonstrated the association of total TILs and the long-term survival were obtained from the published data. Meta-analysis of the included papers reporting DFS and metastasis-free survival in total TILs revealed a pooled HR of 0.88, with a 95 % CI of 0.83–0.98, which is statistically significant (*P* < 0.0001). Total TILs also indicate a long overall survival, but without statistically significant (*P* = 0.08). We also evaluated the prognostic utility of TILs within intraepithelial (iTILs) and stromal compartments (sTILs). The pooled data suggest both iTILs and sTILs were associated with better DFS and cancer-specific survival, estimated HRs in iTILs and sTILs being 0.90 (95 % CI 0.83–0.98) and 0.85 (95 % CI 0.76–0.94), whereas they were not significantly correlated with OS (HR in iTILs 0.90, 95 % CI 0.76–1.06 and HR in sTILs 0.91, 95 % CI 0.77–1.08) (Fig. 3).

**Fig. 4:**
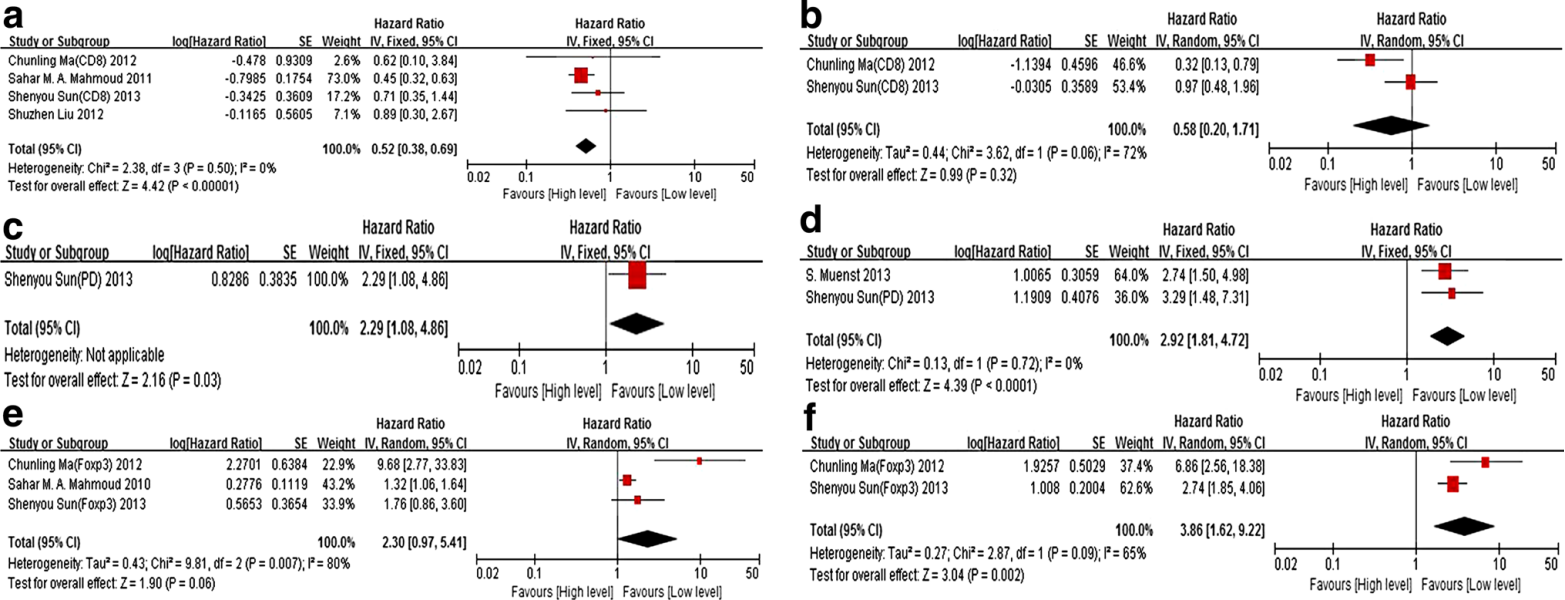
The forest plot of HRs was assessed for association between subtypes of TILs and breast cancer long-time prognosis. **a** CD8^+^ TILs and disease-free survival/cancer-specific survival, **b** CD8^+^ TILs and overall survival, **c** PD-1^+^ TILs and disease-free survival, **d** PD-1^+^ TILs and overall survival, **e** Foxp3^+^ TILs and disease-free survival/cancer-specific survival, **f** Foxp3^+^ TILs and overall survival

In a subgroup analysis, both PD-1^+^ TILs (polled HR 2.92, 95 % CI 1.81–4.72) and Foxp3^+^ TILs (polled HR 3.86, 95 % CI 1.62–9.22) predicted poor overall survival. But there exists no significance for disease-free survival and cancer-specific survival. Breast cancer with high level of CD8^+^ TILs showed a favorable disease-free survival (pooled HR 0.52, 95 % CI 0.30–0.69) (Fig. 4).

Wen-Chung Che’s research illustrated that interleukin-17-producing TILs had a survival influence on breast cancer, this study contained 207 breast cancer patients, the result indicated that IL-17-producing TILs were associated with high grade, hormone receptor (−) subtype, and a poorer survival (disease-free survival 64.0 vs. 87.3 %; HR 2.68; 95 % CI 1.37–5.27; *P* < 0.01) [18]. In addition, there was another small group called γδT TILs in breast cancer. These γδT cells were very common in breast cancer microenvironment, and predicted a better survival (recurrence-free survival: HR 41.69 95 %CI 5.4–321.96; *P* = 4.79 × 10^−8^. Overall survival: HR 44.73 95 %CI 5.79–345.22, *P* = 1.51 × 10^−8^) [19].

### Publication bias

Egger’s tests indicated that there was no evidence of significant publication bias after assessing the funnel plot for the studies included in our meta-analysis (Supplementary Figure 2).

## Discussion

The information about the prognostic and predictive value of TILs in breast cancer is still limited. To our best knowledge, the present meta-analysis is the first study to systematically evaluate the association among TILs with clinical–pathological features and prognostic factors in breast cancer.

### Correlation of TILs with clinicopathological parameters

Total TILs in breast cancer are not strongly associated with clinicopathological characteristics, the previous studies showed that breast cancer with high differentiation, hormone receptor (−) and HER-2 (+) would have a higher level of TILs [13]. But our study did not achieve the similar result; this might be related to very small available raw data and lack of large size of sample research. But PD-1^+^ and Foxp3^+^ subtype TILs highly expressed in hormone receptor (−), HER-2(+) breast cancer, which have a high risk of recurrence and metastasis. However, CD8^+^ TILs as the core of the local immune cells did not have relationship with clinic pathological traits of breast cancer.

### Relationship between TILs and neoadjuvant chemotherapy response

**Fig. 5:**
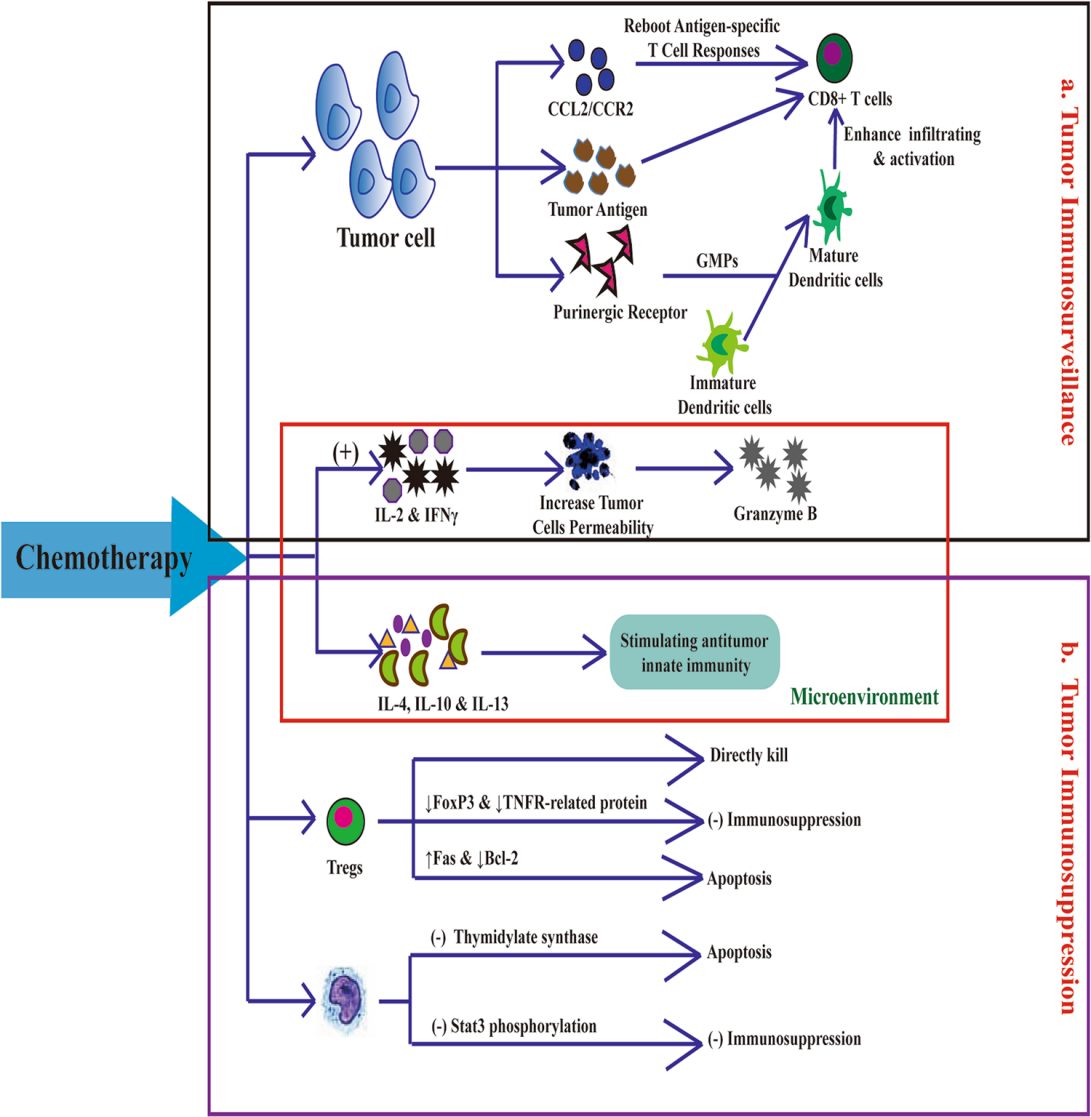
A positive feedback loop between TILs and breast cancer chemotherapy

Recently, the relationship between TILs and the response of breast cancer with neoadjuvant chemotherapy have attracted much attention. Most researches deem that rich of TILs can improve the sensitivity and effect of neoadjuvant chemotherapy. The reasons mainly include (Fig. 5): first, influencing the factors about tumor immunosurveillance. Chemotherapeutic drugs were selected for their direct cytotoxic effects against highly proliferative tumor cells, releasing tumor antigen, ATP and purinergic receptor. Tumor cells expressed HLA-I had higher CD8^+^ T cell infiltration [20]. Besides, ATP-dependent pathway whereby the intratumoral accumulation of granulocyte–monocyte progenitors (GMPs) and inflammatory monocytes facilitates the local differentiation of inflammatory DCs and the activation of T cells against cancer [21]. Chemotherapy promoting chemokine expression in tumor microenvironment affects leukocyte migration, such as CCL2/CCR2 pathway reboots antigen-specific T cell responses [22]. Otherwise, chemotherapy can induce IL-2 and IFNγ secretion to trigger immunogenic cell death, and increase the permeability of tumor cells to granzyme B, thereby rendering them to be susceptible to CTL-mediated lysis even if they do not express the antigen recognized by CTLs [9]. Second, influencing the factors about tumor immunosuppression. Regulatory T lymphocytes (Tregs) and myeloid-derived suppressor cells (MDSCs) are major components of these inhibitory cellular networks [23]. Compared with other immune cell, Tregs have a higher proliferation rate and can be directly “killed” by chemotherapy. Drugs, like cyclophosphamide, can impair Treg-suppressive function by downregulating Foxp3 and glucocorticoid-induced TNFR-related protein. The selective induction of Tregs apoptosis by paclitaxel was attributed to the upregulation of the cell death receptor Fas and downregulation of the antiapoptotic molecule Bcl-2 on Tregs [24]. The antimetabolite like 5-fluorouracil at low doses had also been shown to induce MDSCs apoptosis, this selective effect was explained by a lower expression of thymidylate synthase by MDSCs [25]. Docetaxel and paclitaxel were shown to impair MDSCs suppressive function, predominantly by blocking Stat3 phosphorylation and by promoting MDSC differentiation into M1 macrophages or dendritic cells (DCs). Chemotherapy can also inhibit immunosuppressive cytokines (including IL-4, IL-10 and IL-13) while stimulating antitumor innate immunity [20].

Our study reflected that high-TILs showed a better treatment response of neoadjuvant chemotherapy, but under subtype analysis, we found in hormone receptor (−) breast cancer did not get the same result. This result differed from the Clinical Trial PrECOG0105 [26]. In 2014, American Society of Clinical Oncology (ASCO), Shaveta Vinayak reported that TILs were predictive of response to platinum-based neoadjuvant therapy and were significantly associated with triple-negative breast cancer (TNBC), the causes and possible reasons for this difference may be the chemotherapy agents, eligible studies in our analysis almost used the anthracycline-based chemotherapy.

In 2013, San Antonio Breast Cancer Symposium (SABCS), Sherene Loi reported that TILs were associated with higher pCR rates after neoadjuvant trastuzumab and chemotherapy in early-stage HER2-positive breast cancer [27]. This research gave us a new message that tumor targeted therapy and TILs had a complex relationship, more studies were needed to explore TILs dynamic change with trastuzumab treatment, and the mechanism [28].

Breast cancer is a kind of malignant tumor with high heterogeneity, TILs as a marker for treatment need adequate consideration about the molecular subtypes of cancer and chemotherapy agents. Certain types of breast cancer with appropriate level of chemotherapy enhance local immune reaction and activate cytotoxic lymphocytes. Immune reaction and conventional antineoplastic agents play a double effect on breast cancer and finally improve the therapeutic outcome.

### Impact of TILs on long-term prognosis

Previous studies had shown that higher expression of total TILs suggested a better prognosis of breast cancer, our study also got the similar results. But we found that the value for prognostic implication was not affected by TILs location, both iTILs and sTILs contributed to a favorable survival.

It is worth noting that different TILs subtypes have different results. High level of PD-1^+^ TILs or Foxp3^+^ TILs predicts a poor prognosis. PD-1^+^ TILs or Foxp3^+^ TILs can suppress antitumor immune response and lead to escape immune clearance, so the more the PD-1^+^ TILs or Foxp3^+^ TILs were, the worse the prognosis of patients was [29, 30]. On the contrary, CD8^+^ TILs indicated a good prognosis [31]. These results therefore reflect that lymphocytes in tumor microenvironment can affect the balance of immune response and tolerance, leading to different outcome.

Most studies indicated the association between TILs in pre-treatment and long-term prognosis of breast cancer, but what was it about TILs in residual lesions after neoadjuvant therapy? M. V. Dieci proved that the higher concentration of residual lesions TILs after neoadjuvant therapy predicted a better prognosis, and this is not affected by chemotherapy cycle times [32]. So, TILs dynamics variation may be a good prognostic marker in breast cancer microenvironment.

However, there still exist some limitations in the present meta-analysis. First of all, the literatures in the meta-analysis were based on observational studies, the correctness of the result depended on the accuracy of the original literature research, we therefore formulated the strict inclusion and exclusion standard. Moreover, this study only included the published literature in English and limited quantity literatures, there might exist language bias and study heterogeneity.

In conclusion, our study shows a significant correlation between TILs and clinical traits in breast cancer patients. Higher value of total TILs not only predicts neoadjuvant chemotherapy response, but also implies a better prognosis, whereas some subtypes of TILs, like PD-1^+^ TILs and Foxp3^+^ TILs, are not amity with breast cancer and predict an unfavorable outcome. But for hormone receptor (−) breast cancer, neoadjuvant chemotherapy agent may affect the TILs infiltrating, and lead to a different therapeutic effect. So TILs should be monitored in breast cancer patients for rational stratification and adjusting the treatment strategy, further meticulous and deep researches about TILs and breast cancer are also needed.

## Electronic supplementary material

Below is the link to the electronic supplementary material.

Supplementary material 1 Supplementary Fig. 1 The forest plot of RRs was assessed for association between TILs and breast cancer clinicopathological features. (**a**) Total TILs, (**b**) CD8^+^ TILs, (**c**) PD-1^+^ TILs, (**d**) Foxp3^+^ TILs (TIFF 834 kb)

Supplementary material 2 Supplementary Fig. 2 The funnel plots. (**a**) TILs and breast cancer short-term outcome (neoadjuvant chemotherapy pCR rate), (**b**) total TILs and breast cancer long-term prognosis (TIFF 5423 kb)
