# Supplementary figures and images for: Prognostic and predictive value of tumor-infiltrating lymphocytes in breast cancer: a systematic review and meta-analysis
Source: Clin Transl Oncol. 2015 Oct 12;18:497–506. doi: 10.1007/s12094-015-1391-y (PMC4823351; doi:10.1007/s12094-015-1391-y)

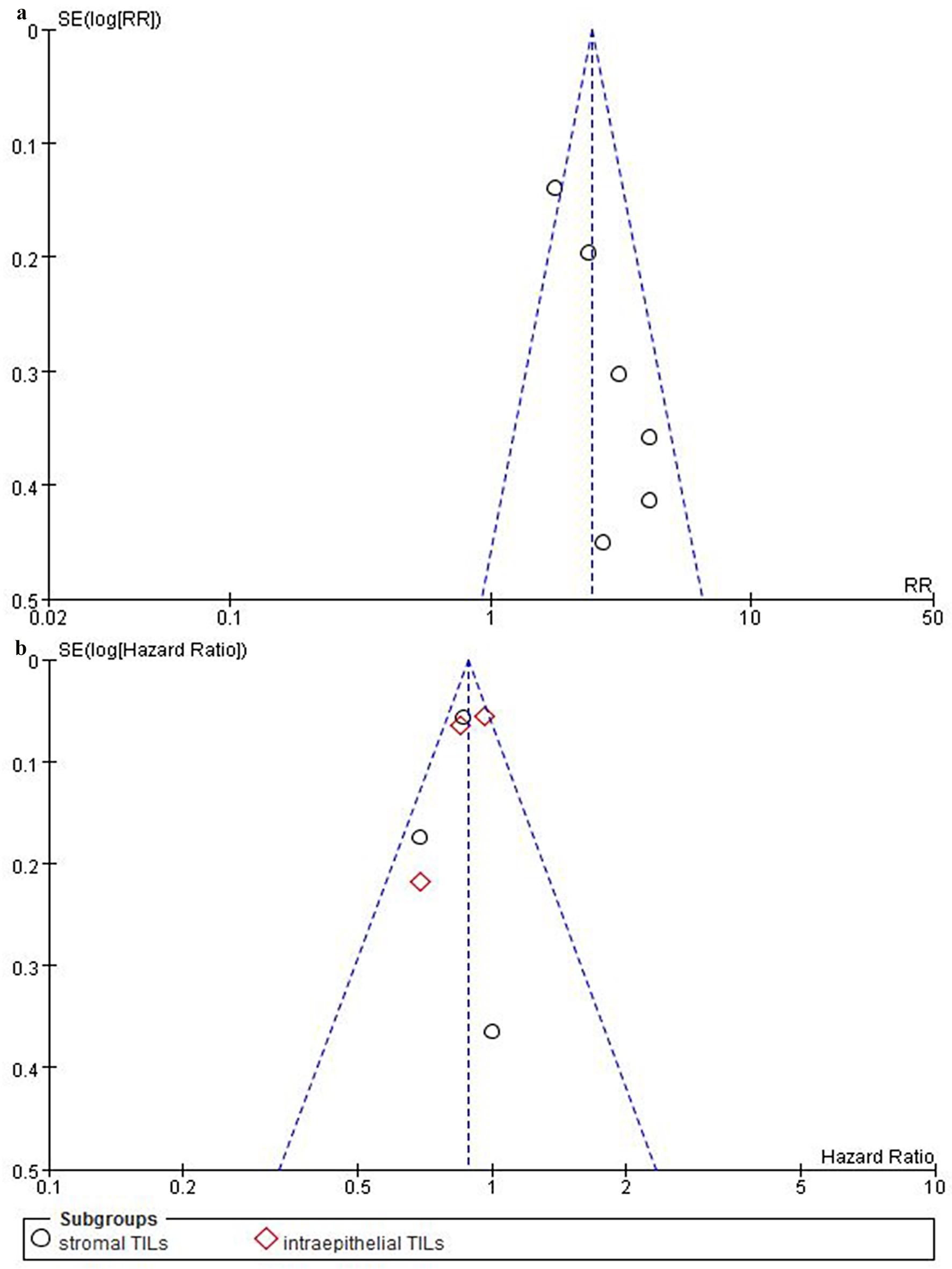

Supplement: Supplementary file 1 — Supplementary material 1 Supplementary Fig. 1 The forest plot of RRs was assessed for association between TILs and breast cancer clinicopathological features. (a) Total TILs, (b) CD8+ TILs, (c) PD-1+ TILs, (d) Foxp3+ TILs (TIFF 834 kb) [file 12094_2015_1391_MOESM1_ESM.tif]

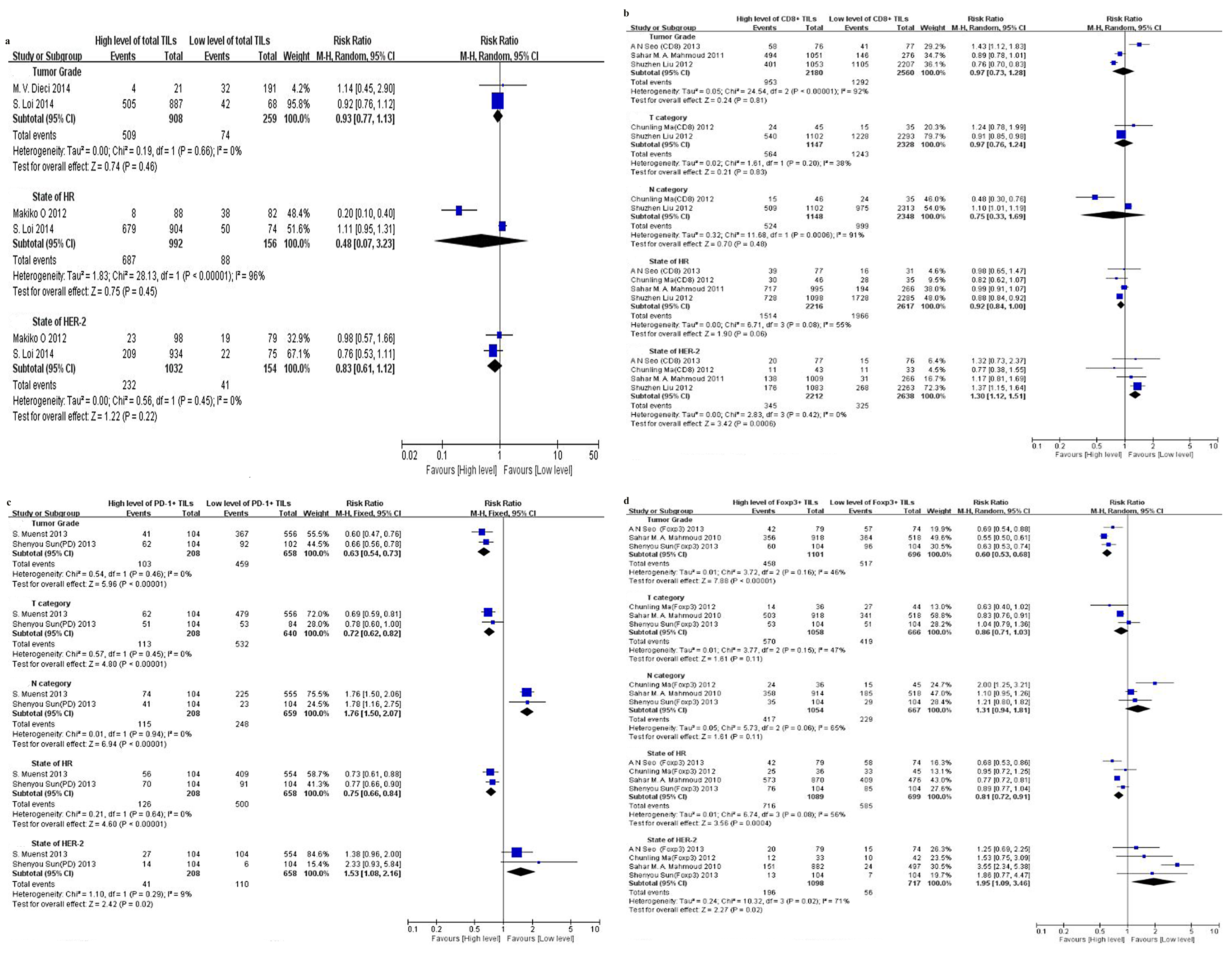

Supplement: Supplementary file 2 — Supplementary material 2 Supplementary Fig. 2 The funnel plots. (a) TILs and breast cancer short-term outcome (neoadjuvant chemotherapy pCR rate), (b) total TILs and breast cancer long-term prognosis (TIFF 5423 kb) [file 12094_2015_1391_MOESM2_ESM.tif]
